# Efficiency of Dexamethasone for Treatment of Vasogenic Edema in Brain Metastasis Patients: A Radiographic Approach

**DOI:** 10.3389/fonc.2019.00695

**Published:** 2019-07-30

**Authors:** Tanja Schroeder, Paul Bittrich, Clara Noebel, Jan Felix Kuhne, Julian Schroeder, Gerhard Schoen, Jens Fiehler, Helge C. Kniep, Susanne Gellißen

**Affiliations:** ^1^Department of Diagnostic and Interventional Neuroradiology, University Medical Center Hamburg-Eppendorf, Hamburg, Germany; ^2^Department of Radiology, Schoen Klinik Hamburg Eilbek, Hamburg, Germany; ^3^Department of Neurology, University Medical Center Hamburg-Eppendorf, Hamburg, Germany; ^4^Department of Medical Biometry and Epidemiology, University Medical Center Hamburg-Eppendorf, Hamburg, Germany

**Keywords:** brain metastases, brain metastasis, glucocorticoids, dexamethasone, vasogenic edema

## Abstract

**Background and Purpose:** To date, imaging studies quantifying the amount of vasogenic edema reduction (VE) in patients with brain metastases (BM) treated with glucocorticoids (GC) have included a very limited number of patients and showed ambiguous results. Here, we aim to determine the radiological effect of GC on VE in BM patients in a large cohort with multiple primary tumor entities in a cross-sectional approach.

**Materials and Methods:** This monocentric retrospective study includes 299 patients first-ever diagnosed with 2,759 intra-axial BM on the respective MRI. 126/299 patients received GC prior to MRI due to mass effect of edema on cranial CT scan and clinical symptoms (GC-pos) and 173 patients did not (GC-neg). GC dose was documented in 85/126 patients. All BM and their respective VE were semi-automatically segmented on post-contrast T1-weighted images.

**Results:** VE volumes were higher in GC-pos compared to GC-neg (*p* = 0.009) and did not correlate with GC dose. Multivariate linear regression analysis with interaction terms on the assumption that BM volume and BM number influence the probability of GC administration shows that large and higher numbers of BM under GC treatment generate less VE than without (*p* < 0.001 and *p* = 0.038, respectively). The primary tumor type and total BM volume did not influence VE volume.

**Conclusion:** Use of GC is especially effective for treatment of VE formation in patients with larger and multiple BM regardless of primary tumor type and dosage. However, based on the present data a direct causative relationship between GC and VE cannot be proven.

## Introduction

If the size of a brain metastasis (BM) exceeds a certain threshold, occurrence of a vasogenic cerebral edema (VE) due to tumor-induced disruption of the blood-brain-barrier is a well-known phenomenon (1). During this process, fluid leakage from defective capillaries into the extracellular space contributes significantly to overall mass effect. In this setting, application of systemic (oral or intravenous) glucocorticoids (GC) to reduce BM capillary permeability is the first-line treatment (2, 3). Dexamethasone has a lower mineralocorticoid activity and a longer plasma half-life than other synthetic GC and is thus the most commonly used drug (3). There is very limited study data on dosage of dexamethasone. Practice guidelines from a systematic review including two studies recommended a starting dose of 4–8 mg/day for patients with mild symptoms and 16 mg/day for those with moderate and severe symptoms attributed to mass effect from BM and VE (4–6).

Prior reports on the radiographic effect of GC in BM patients showed ambiguous results: whereas two studies demonstrated reduced VE size after GC administration in 3 and 13 BM patients, three other studies were not able to detect significant differences in VE volume in 4, 7, and 4 BM patients (7–11). Most patients presenting with clinical symptoms due to mass effect of BM receive GC immediately in the emergency room or even before referral to our academic medical center. It is thus hardly feasible and due to the critical nature of the condition ethically questionable to conduct a randomized trial evaluating the radiographic effect of GC on VE. A different approach was therefore used in our study: we included patients who received GC prior to diagnosis of BM confirming MRI (GC-pos) or not (GC-neg) and adjusted for GC administration afterwards.

Here, we sought to further evaluate the impact of GC on VE volume in BM patients and examine whether a relevant correlation to BM size, number of BM, primary tumor entity, and dose of GC exists. We hypothesized that administration of GC is especially effective in large BM and at high doses.

## Materials and Methods

### Study Cohort

This single center retrospective cross-sectional study was conducted in compliance with the local ethics committee (Ethik-Kommission der Aerztekammer Hamburg, WF-018/15) under the waiver of informed consent.

**Figure 1:**
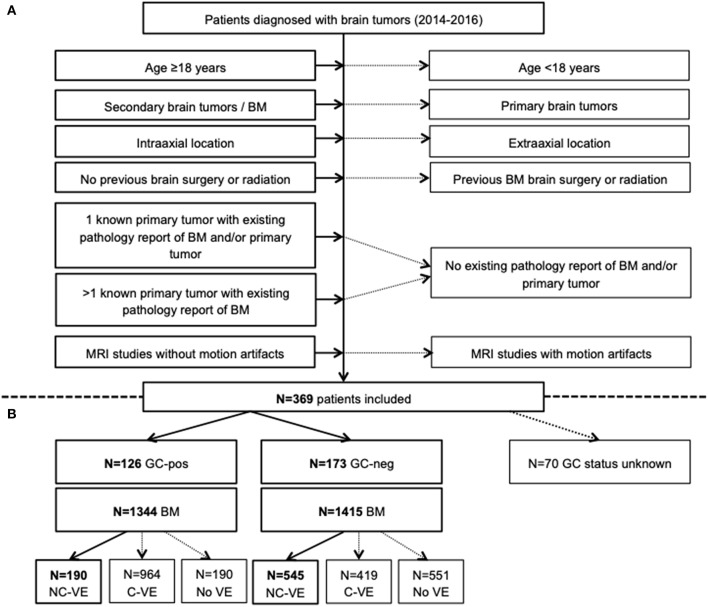
Flow-chart summarizing patient selection in the study **(A)** and distribution of patients who received GC (GC-pos) and who did not (GC-neg) with corresponding number of brain metastases (BM) and edema (VE, **B**). Only VE without connection to an adjacent VE (NC-VE) were segmented and included in final analysis (thick boxes). BM, brain metastases; C-VE, confluent vasogenic edema; GC-neg, patient did not receive glucocorticoids; GC-pos, patient received glucocorticoids; MRI, magnetic resonance imaging; NC-VE, non-confluent vasogenic edema; y, years.

Over a three-year period, we found 369 patients in whom cranial MRI was performed and first diagnosis of intra-axial BM was made. MRI was either performed following a suspicious head CT in patients with acute neurologic deficits or during routine diagnostic work-up in less symptomatic or asymptomatic patients. Those with acute illness due to tumor mass effect and surrounding edema on CT routinely received GC (GC-pos, *n* = 126 patients). GC dosage was at the discretion of the treating physician in the emergency department. In *n* = 173 cases, no GC were administered prior to MRI since BM were initially diagnosed here. In 70/369 patients it was unknown if they received GC prior to imaging or not. Overall, we included 299 patients with known GC status and their respective MRI at the time of first diagnosis of BM (Figure 1A).

Afterwards, electronic chart review of eligible cases was performed. Type of primary neoplasm and detailed information about GC treatment (dose, duration, and drug name) were further collected. GC doses of different drugs were adjusted by multiplying dose by relative GC potency that is 30 for dexamethasone and 4 for prednisolone (12).

### MRI Study Protocol

Mri was performed using a 1.5 Tesla (Magnetom® Sonata, Siemens Healthcare, Erlangen, Germany; Magnetom® Symphony, Siemens Healthcare, Erlangen, Germany, and Magnetom® Avanto, Siemens Healthcare, Erlangen, Germany) in 271 patients or a 3 Tesla scanner (Magnetom® Skyra, Siemens Healthcare, Erlangen, Germany; Ingenia, Philips Medical Systems, Best, The Netherlands) in 28 patients. Imaging protocol included native axial fluid-attenuated inversion recovery in 249 or T2-weighted turbo spin echo imaging in 50 patients (T2w). Following weight-adjusted intravenous Gadolinium injection, axial T1-weighted spin echo with flow compensation and three-dimensional T1w gradient echo sequences were acquired in 75 and 224 patients, respectively (T1w+). Sequence parameters varied among the different scanners and were published before (13).

### Image Analysis

First, all 2759 BM were semi-manually segmented on each T1w+ slice with Analyze Software System 11.0 (Biomedical Imaging Resource, Mayo Clinic, Rochester, NY, USA) (14). In a second step, each BM was determined to be either VE-negative (no edema visible) or VE-positive (edema visible). In latter cases, we distinguished between BM with a non-confluent VE (no connection to an adjacent VE of another BM with following segmentation on each T2w slice) and a confluent VE (connection to an adjacent VE with no segmentation on T2w, Figure 1B). Thirdly, T1w+ images were automatically co-registered to the 1 mm Montréal Neurological Institute (MNI) standard space using the Oxford Centre for Functional Magnetic Resonance Imaging of the Brain Software Library 5.0 (Analysis Group, Oxford, UK) linear (affine) registration tool. Thus, BM location could be determined based on anatomical regions defined by the MNI atlas.

Accuracy of both BM/VE masks and correct registration of T1w+ images to the MNI space was evaluated and corrected if applicable by two independent readers (S. G. and T. S. with 12 and 4 years of experience in Neuroradiology, respectively).

### Statistical Analysis

Statistical analysis was conducted using R software (version 3.4.4; The R Foundation for Statistical Computing, Vienna, Austria) and IBM SPSS Statistics® (version 20, IBM® 2011, Armonk, NY, USA). Univariate differences between GC-pos and GC-neg patients in Table 1 were calculated using either Mann-Whitney-U-test (age at first diagnosis of cancer or BM and latency in between, number of BM) or Pearson Chi-square test (distribution of sex and primary tumor entities).

**Table 1:** Clinical and tumor-related features of GC-positive and GC-negative patients.

**Characteristic**	**GC-pos**		**GC-neg**		***P***
	***N*** **=** **126**		***N*** **=** **173**		
Age as median (IQR)					
At diagnosis of primary tumor (years)	61 (52–68)		61 (51–70)		0.792
At diagnosis of BM (years)	63 (56,75–71)		62.5 (53–72)		0.406
Latency (months)	12 (0–34.75)		12 (0–33.5)		0.312
Sex	*N*	%	*N*	%	0.452
Female	66	52.4	83	47.7	
Male	60	47.6	90	52.3	
Number of BM as median (IQR)	3 (1–11)		2 (1–6)		0.062
Primary tumor	*N*	%	*N*	%	0.437
Lung cancer	52	41.3	85	49.1	
Non-small cell	34	27.0	61	35.2	
Small cell	17	13.5	24	13.9	
Unknown	1	0.8	–	–	
Genitourinary cancer	20	15.9	20	11.6	
Kidney	6	4.8	8	4.6	
Prostate	5	4	5	2.9	
Urothelium	5	4	1	0.6	
Ovary	2	1.6	2	1.2	
Testicles	2	1.6	2	1.2	
Uterus	–	–	2	1.2	
Breast cancer	18	14.3	18	10.4	
Skin cancer	11	8.7	20	11.6	
Melanoma	11	8.7	18	10.4	
Merkel cell carcinoma	–	–	1	0.6	
Squamous cell carcinoma	–	–	1	0.6	
Gastrointestinal cancer	14	11.1	17	9.8	
Colon	6	4.8	5	2.9	
Rectum	2	1.6	5	2.9	
Esophagus	2	1.6	4	2.3	
Gastroesophageal junction	2	1.6	2	1.2	
Stomach	–	–	1	0.6	
Neuroendocrine	1	0.8	–	–	
Cholangiocellular carcinoma	1	0.8	–	–	
Cancer of unknown primary	6	4.8	10	5.8	
Sarcoma	5	4	2	1.2	
Head and Neck	–	–	1	0.6	
Thyroid	–	–	1	0.6	

*BM, brain metastases; GC-neg, patients who did not receive glucocorticoids; GC-pos, patients in whom glucocorticoids were given*.

In order to reduce skewness and obtain a near-Gaussian distribution the following variables were transformed prior to analysis: (a) for BM and VE volumes (originally in mm^3^), the logarithm of their cube root was calculated and (b) GC doses were logarithmized.

To calculate differences between VE volumes of GC-pos and GC-neg patients, a random intercept model was run with status of GC application as fixed and patient identifier as random effect. The patients in whom GC dose was documented (patient identifier as random factor) were included in another random intercept model calculating the relationship between daily dose of GC (fixed factor) and VE size.

Furthermore, multivariate linear regression analyses were performed in order to determine if clinical (primary tumor entity as fixed effect) and BM-related factors (volume of the single BM, volume of all BM, number of BM, and GC application status as fixed effects) influence VE size (dependent variable); first without and afterwards with interaction terms. We used interaction terms on the assumption that the variables GC application status, volume of single, and number of BM have not only an additive but also a simultaneous effect. BM volume and BM number influence the probability of GC administration; i.e., these variables interact with each other. Patient identifier was set as random factor. In addition, effect plots were created for the variables that have a significant impact on VE volume.

A *p* < 0.05 was considered significant. If not otherwise indicated, data are given as median (interquartile range).

## Results

### Descriptive Statistics

Table 1 shows distribution of clinical (age, sex) and tumor-related (number of BM per patient, primary tumor entities, and histological subtypes) features across GC-pos and GC-neg patients. Univariate analyses confirm an equal distribution between both groups.

### GC Statistics and VE Volumes

Of 126 GC-pos patients, GC dose was documented in 85 patients with 841 BM. 81 (95.3%) patients took dexamethasone only, 2 (2.4%) patients received prednisolone only, and 2 (2.4%) patients took prednisolone + dexamethasone. Overall, patients took a cumulative GC dose with a relative GC potency of 960 (360–1560) over a period of 3 (2–4) days resulting in a GC dose of 360 (240–480) per day.

**Figure 2:**
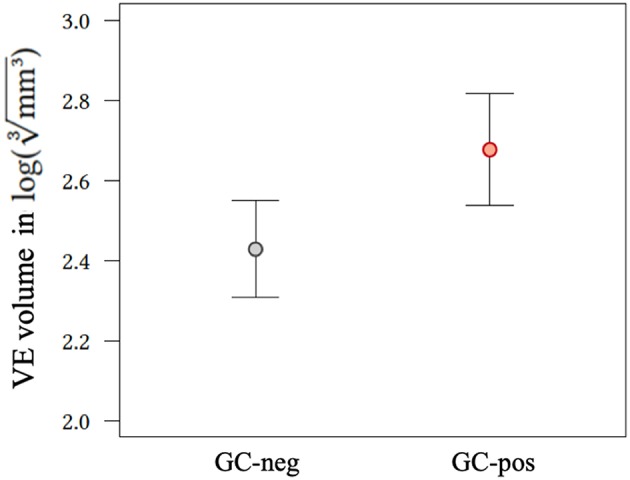
Effect plot of application status of glucocorticoids on VE volume. GC-neg, patients who did not receive glucocorticoids; GC-pos, patients in whom glucocorticoids were given; VE, vasogenic edema.

Random intercept model showed larger VE volumes for GC-pos patients compared to GC-neg ones [2.68 (CI, confidence interval, 2.36–2.50) vs. 2.43 (CI 2.61–2.75); *p* = 0.009, Figure 2].

Random intercept model calculating the relationship between daily dose of GC and VE volume revealed no significant correlation (0.142, confidence interval −0.102–0.386, *p* = 0.259).

### Multivariate Analyses

**Figure 3:**
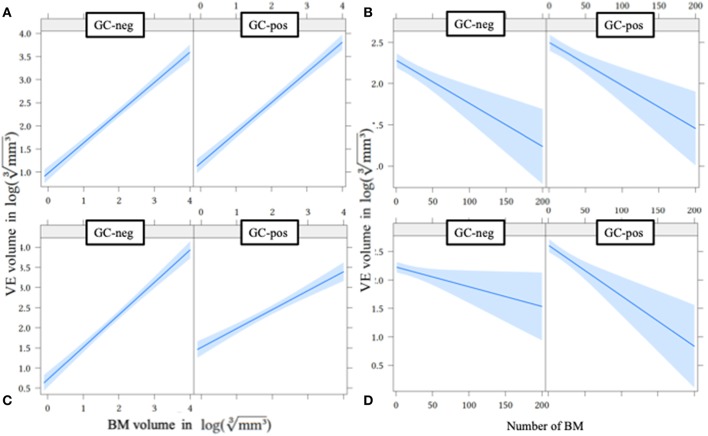
Effect plot of metastases volume **(A,C)** and number of metastases **(B,D)** on VE volume without **(A,B)** and with **(C,D)** interaction terms. BM, brain metastases; GC-neg, patients who did not receive glucocorticoids; GC-pos, patients in whom glucocorticoids were given; VE, vasogenic edema.

In multivariate linear regression analysis including primary tumor entities, volume of single BM, volume of all BM, number of BM, and GC application status (without interaction terms), primary tumor groups and the volume of all BM showed the highest *p*-values and were hence removed from the model. Volume of single BM (0.653, CI 0.590–0.717, *p* < 0.001), number of BM (−0.005, CI −0.008–0.003, *p* < 0.001), and GC application status (also see Figure 2) showed a significant influence on VE volume (Table 2 and Figures 3A,B).

**Table 2:** Multivariate linear regression analysis in order to determine if volume of the single BM, volume of all BM, number of BM, and GC application status influence VE size without and with interaction terms.

	**Coefficient**	**95% CI**	***P***
**WITHOUT INTERACTION TERMS**			
(Intercept)	1.144	0.984–1.302	<0.001
GC-pos	0.218	0.101–0.334	<0.001
Single BM volume	0.653	0.590–0.717	<0.001
Number of BM	−0.005	−0.008–(−0.003)	<0.001
**WITH INTERACTION TERMS**			
(Intercept)	0.822	0.622–1.020	<0.001
GC-pos	0.966	0.652–1.271	<0.001
Single BM volume	0.805	0.719–0.891	<0.001
Number of BM	−0.003	−0.007–0.000	0.034
GC-pos^*^Single BM volume	−0.334	−0.459–(−0.205)	<0.001
GC-pos^*^Number of BM	−0.005	−0.010–0.000	0.038

*BM, brain metastases; CI, confidence interval; GC-pos, patients in whom glucocorticoids were given*.

After including interaction terms (GC-pos^*^single BM volume and GC-pos^*^number of BM), all effects remained significant, but showed a growing dependence from GC application status (Table 2 and Figures 3C,D): if GC are administered, VE sizes of larger and multiple BM were proportionally smaller compared to VE volumes of smaller and singular BM (less steeply rising graphs for GC-pos (Figure 3C; slope is 0.805) compared to GC-neg (Figure 3D; slope is 0.805+(−0.334) = 0.471). This finding suggests that (after adjustment for BM size and the number of individual BM) BM generate less VE under GC treatment than without. This effect gets more relevant with increasing volume and number of BM.

## Discussion

In this study we aimed to determine the radiological impact of GC on VE volume and its contributing factors in 299 patients at time of first diagnosis of BM at our university medical center. All patients underwent cranial MRI, either receiving GC prior to MRI due to mass effect on emergency head CT scan (*n* = 126) or not (*n* = 173). Our results indicate that patients with large and higher numbers of BM benefit most from GC therapy since their VE volumes were proportionally smaller compared to patients in whom no GC were administered. There was no association to the primary tumor type or dose of GC.

The decision of giving GC was at the discretion of the treating physician in this study. In fact, indication and dosage of GC is far from being highly standardized because there is a very limited number of randomized trials and only one meta-analysis with level III evidence addressing this issue (4–6). We therefore think that our finding that VE volume is independent of GC dose may be of limited value and should be validated in a prospective, randomized trial.

Our results indicate that—after adjustment for volume and the number of individual BM—treatment with GC leads to decreasing VE volumes in large and higher numbers of BM. In contrast, we found 3 former studies propagating no effect of GC administration on VE size (8, 10, 11). This might be due to measurement errors for small BM (according to the formula for volume of a sphere, little changes of diameter lead to significant volume changes) or GC may be ineffective until the VE exceeds a certain volume threshold what we consider less likely.

In our cohort, we found VE size being independent from the tumor type. Interestingly, prior animal studies demonstrated different mechanisms in BM outgrowth of different primary neoplasms: Kienast et al. showed that melanoma cells prefer recruitment of preexisting parenchymal vessels whereas pulmonary cancer cells mainly induce neoangiogenesis per vascular endothelial growth factor (VEGF) secretion (15). Since VEGF plays an important role in the development of VE in BM patients and is inhibited by GC (see below), the primary tumor entity might nevertheless influence GC efficacy.

The mechanism of action of GC in BM patients is related to the downstream genomic (mostly transcriptional) and, to a lesser extent, direct (non-genomic) effects after passing the cellular membrane and binding of GC to the cytoplasmatic GC receptor. Those signaling effects are thought to include inhibition of the capillary permeability-mediating VEGF pathway, modulation of key tight-junction proteins, and increased synthesis of cytoskeleton-stabilizing protein vascular-endothelial cadherin; all leading to a reduction of porosity of disrupted blood-brain-barrier (2, 16–19). Besides the positive impact on VE volume, GC have side effects especially like hyperglycemia (“steroid diabetes” which was shown to shorten overall survival in glioblastoma patients) with Cushing's syndrome (20). Hence, our finding that GC are more effective in severe cases emphasizes that dosage adjustments based on symptom severity are essential and are supported by MRI based volumetry in this study (4, 6, 20).

Our study has several limitations. First, patients with larger and more individual metastases are more likely to receive GC treatment. This results in a significant selection bias for GC positive patients as indicated by significantly higher VE (Figure 2) and BM volumes (Figure 3A) in the GC-pos compared to the GC-neg cohort. To account for this bias we adjusted for volume and the number of individual BM in our multivariate analysis. Second, we included only a single time point (the initial imaging study) and therefore could not evaluate the direct individual effect of GC administration on VE size changes. Thus, a direct causative relationship between GC and VE cannot be shown due to a lack of baseline or follow-up imaging in our collective. However, VE volumes in follow-up studies could have been biased by chemotherapy or radiation regimens hence requiring a prospective randomized trial accounting for all those factors. Third, we did not record the patients therapies prior to diagnosis of BM, especially if they were on immunotherapeutic regimens what would have potentially influenced VE volume. However, we think that only a minority of patients would have been affected since only a small number of drugs were available in selected patient populations in Germany at the time of the study.

In conclusion, our results suggest that administration of GC is especially effective in VE from patients with larger and multiple BM regardless of primary tumor type and dosage. Due to significant adverse effects of GC there is no indication to prescribe GC for asymptomatic patients and it should be checked regularly if a lower GC dose is equally sufficient.

## Ethics Statement

This study was conducted in compliance with the local ethics committee (Ethik-Kommission der Aerztekammer Hamburg, WF-018/15) under the waiver of informed consent.

## Author Contributions

TS, PB, GS, and SG: conceptualization. TS, PB, CN, JK, JS, GS, JF, HK, and SG: investigation. TS, PB, GS, and SG: methodology.HK and SG: software. TS and JS: writing original draft. TS, PB, CN, JK, JS, GS, JF, HK, and SG: writing, reviewing and editing.

### Conflict of Interest Statement

The authors declare that the research was conducted in the absence of any commercial or financial relationships that could be construed as a potential conflict of interest.
